# A repeated cross-sectional study of the association of community health worker intervention with the maternal continuum of care in rural Liberian communities

**DOI:** 10.1186/s12884-023-06162-8

**Published:** 2023-12-07

**Authors:** Sam Blizzard, Mardieh Dennis, Marion Subah, Bentoe Zoogley Tehoungue, Romax Zizi, John D. Kraemer, Emily White, Lisa R. Hirschhorn

**Affiliations:** 1 Feinberg School of Medicine, Northwestern University, Chicago, IL USA; 2 Last Mile Health, Monrovia, Liberia; 3 Ministry of Health, Monrovia, Liberia; 4 Department of Health Management and Policy, Georgetown University School of Health, Washington, DC USA; 5 Department of Medical Social Sciences and Ryan Family Center for Global Primary Care, Havey Institute for Global Health, Feinberg School of Medicine, Northwestern University, Chicago, IL USA

**Keywords:** Maternal continuum of care, Community health worker, Rural, Liberia

## Abstract

**Background:**

The maternal continuum of care (CoC) (antenatal care, facility-based delivery, postnatal care) is critical to maternal and neonatal health and reducing mortality, but completion in rural areas of low- and middle-income countries is often limited. We used repeated cross-sectional household surveys from a rural Liberian county to explore changes in rates of completion of all steps and no steps in the maternal CoC after implementation of the National Community Health Assistant Program (NCHAP), a community health worker (CHW) intervention designed to increase care uptake for families over five kilometers from a facility.

**Methods:**

We analyzed repeated cross-sectional household surveys of women aged 18–49 served by NCHAP in Rivercess County, Liberia. We measured survey-weighted, before-to-after implementation difference in completion of all steps and no steps in the maternal CoC. We used multivariable regression to explore covariates associated with completion rates before and after NCHAP implementation.

**Results:**

Data from surveys conducted at three timepoints (2015, n = 354; 2018, n = 312; 2021, n = 302) were analyzed. A significant increase in completing the full maternal CoC (2015:23.6%, 2018:53.4%, change:29.7% points (pp), 95% confidence interval (CI) [21.0,38.4]) and a decrease in completing no steps in the CoC (2015:17.6%, 2018:4.0%, change: -12.4pp [-17.6, -7.2]) after implementation of NCHAP were observed from 2015 to 2018, with rates maintained from 2018 to 2021. Living farther from a facility was consistently associated with less care across the continuum. Following implementation, living in a motorbike accessible community was associated with completing the CoC while living in a mining community was negatively associated with omitting the CoC. Household wealth was associated with differences in rates pre-NCHAP but not post-NCHAP.

**Conclusions:**

Following NCHAP implementation, completion rate of the full maternal CoC in Rivercess County more than doubled while the rate of completing no steps in the continuum fell below 5%. These rates were sustained over time including during COVID-19 with reduced differences across wealth groups, although far distances remained a risk for less care. CHW programs providing active outreach to remote communities can be important tools for improving uptake of interventions and reducing risk of no formal care during and after pregnancy.

**Supplementary Information:**

The online version contains supplementary material available at 10.1186/s12884-023-06162-8.

## Background

Ensuring uptake of evidence-based care throughout pregnancy, delivery and post-partum periods is critical to the health of mothers and their neonates [1]. Receipt of these components of health delivery across the maternal continuum of care (CoC) has been associated with reductions in maternal and neonatal morbidity and mortality [2]. Updated recommendations by the World Health Organization (WHO) include eight antenatal contacts, delivery by trained birth attendants in a hygienic and adequately-supplied facility, and four postnatal contacts [3, 4]. Despite these recommendations, access to and completion of the full maternal continuum of care remains limited, especially for low- and middle- income countries in Sub-Saharan Africa [5, 6].

Studies examining factors associated with rates of completion of the triad of the full maternal continuum of care in countries in Asia and Africa found that increased household wealth [7–13], maternal education [7–11, 14, 15], increased maternal age [7, 10, 14, 16], birth order [7–11], mass media exposure [8–10], and living in urban areas [7, 9, 14] were associated with completion of the maternal continuum of care. In 2012, Kenny et al. found that Liberians who lived farthest from health facilities had lower rates of antenatal care (ANC), facility-based delivery (FBD), and postnatal care (PNC) than those who lived closer to a facility (odds ratios for farthest quartile ranged from 0.04 to 0.44 compared to reference quartile of those closest to a facility) [17].

Community health worker (CHW) interventions have been found to contribute to increased rates in various steps of the maternal continuum of care individually, including antenatal care [18–20], facility-based delivery [19, 20], as well as post-natal newborn interventions including breastfeeding [18–21], skin-to-skin contact [22], umbilical cord care [18, 20], and child immunization [19, 23]. CHWs were also associated with increased rates of antenatal care and presence of skilled birth attendants, but not completion of the full continuum of care [21]. However, fewer studies examined rates of postnatal care access, and have called for more community-based interventions in Africa to match the success of similar programs implemented in regions such as south Asia [20].

In Liberia, following the 2014 Ebola epidemic, major shifts to national health care strategy resulted in the 2016 launch and subsequent implementation of a nationwide community health assistant (CHA) program. This program arose from partnerships with non-governmental organizations including Last Mile Health, [24, 25], International Rescue Committee, Partners In Health, UNICEF, and others, with funding from multiple sources including Co-Impact, Global Fund, World Bank, and USAID. The first community health worker programs that would eventually become the NCHAP began coverage in 2012 in Grand Gedeh County and began expanding to Rivercess County in 2014. Following the Ebola pandemic, efforts to grow a redesigned community health worker program began in 2015. Now, the National Community Health Assistant Program (NCHAP) reaches all counties and almost 80% of communities located five or more kilometers from the nearest health facility, and those communities contain approximately 29% of the total Liberian population [24, 26]. The NCHAP has been associated with improved access and uptake of childhood treatment of fever, diarrhea, acute respiratory infections, and malaria [27], as well as increases in facility-based delivery for rural communities [25]. Their efforts to reach remote communities are based on Liberia’s high proportion of rural residents who face lengthy travel to health care. According to 2013 DHS data, 65% of Liberians walk to their nearest health facility, and 27% travel 60 or more minutes by their usual mean of transportation to reach their nearest health facility [28]. Country-level data has shown mixed improvements in pregnancy and neonatal metrics over the period of NCHAP implementation. Demographic and Health Survey (DHS) data shows that percentage of Liberian women receiving four or more prenatal care visits increased from 78 to 87% between 2013 and 2019 and delivering in a facility increased from 56 to 80%. However, over the same period, infant mortality rates in Liberia have increased from 54 to 63 deaths per 1,000 live births [29]. The association of CHAs with improved access to and completion of the full maternal continuum of care in rural communities is less known, especially following the recent COVID-19 pandemic which started in March 2020 in Liberia. The pandemic has been estimated to have caused drops of 2%, 4%, and 4.5% in Liberian rates of antenatal care, facility-based delivery, and postnatal care, respectively, from March to July 2020 [30].

In this study, we examined if implementation of the National CHA Program was associated with increased completion of the maternal continuum of care in Rivercess County. We also explored factors associated with women’s completion of steps in the maternal continuum of care from antenatal through postnatal care. Understanding if CHW programs contribute to completion of the continuum of care can help inform work for future community-based interventions targeting completion of the maternal continuum of care including strategies needed to address individual and structural factors associated with care gaps that persist in maternal and neonatal health care access and delivery.

## Methods

### Study sites

The study was completed in Rivercess County, in which the National CHA Program implementation is supported by Last Mile Health. Rivercess is a majority-rural county located along Liberia’s central coast with population estimate of 71,000, divided into six health districts [31]. The CHA program in Rivercess County was implemented simultaneously across the districts in August of 2015. Implementation included recruitment, training, and deployment of CHAs back into their communities utilizing a five S’s approach: selection, skills, supervision, salary, and supplies [26]. CHAs are trained in facilitating access to and receipt of maternal and newborn health services through a number of methods including home-based education, scheduling of facility-based deliveries, partnerships with trained traditional midwives, and regular screenings and referrals to higher levels of care as necessary. They also receive practical and behavioral training on and provide care regarding family planning (condoms, contraceptive pills, and natural family planning), pregnancy (identifying and treating common problems and warning signs, promoting health care services), and postnatal care (initiation of breastfeeding, care of normal babies, and identification of danger signs). LMH also provides food and transport incentives to pregnant mothers supported by the program. Currently, CHAs are paid $70 per month without compensation for referrals to health facilities, as previously true prior to 2016. In 2016, a coalition of community health stakeholders and partners developed revised the Revised National Community Health Services Policy, which introduced new renumeration plans for CHAs, a standardized approved Ministry of Health training package, supervision by a health profession cadre of Community Health Services Supervisors (CHSSs), refresher trainings for CHAs, and provision of community level supplies and commodities. Since then, a systems-level approach has been utilized to standardize data collection and improve implementation practices to best achieve target outcomes. Following NCHAP implementation, all rural communities in Rivercess were receiving the program. In 2018, the average number of CHAs in Rivercess County was 246 with 22 supervisors; in 2021 there were on average 271 CHAs and 31 supervisors. Full details are located in Luckow, et al. [25].

### Data sources

The study utilized existing data collected over time from a repeated cross-sectional household survey of communities served by the CHA program in Rivercess. The survey was adapted from the Liberian Demographic and Health Survey, and included household information (members, household material composition, cooking features, hygiene features, animals) in addition to questions for eligible women regarding schooling, length of time in community, pregnancy and birth history, breastfeeding practices, family planning, child illness treatment, and child vaccine receipt [32]. We included surveys completed before NCHAP implementation in 2015 (data collected March to May), and then post-implementation surveys in 2018 (May to June) and 2021 (October to November)– see Additional Files 1–3 for full survey texts.

### Survey sampling methods

The household survey methods are described in detail elsewhere [27, 33]. Briefly, households in communities located five or more kilometers from the nearest primary care health center were selected at random through a population-representative two-stage cluster-sample that first randomly selected communities and then households within selected communities. Trained enumerators fluent in both Liberian Vernacular English and Bassa administered the survey and were subject to regular quality assurance checks. All six Rivercess County districts were surveyed at all three timepoints.

### Study population

Women who completed the survey between the ages of 18 and 49 who lived in a community at least five kilometers from the nearest health care facility and who reported a live birth within the last two years of the time surveyed were included. The 2021 survey included 15-17-year-olds, but they were excluded to maintain consistency with the previous surveys Births were included if they occurred within 2 years of the date of the survey (for 2015 survey: March 2013 – May 2015; for 2018 survey: May 2016 – June 2018; for 2021 survey: October 2019 – November 2021), and if a women had more than one birth in the past 2 years, only the most recent birth was included. The two year criteria was chosen to match Demographic and Health Survey (DHS) and Multiple Indicator Cluster Survey (MICS) standards and allow time to ensure that the entire maternal continuum of care from antenatal care to postnatal care took place entirely before (baseline survey) or entirely after (follow up surveys) implementation of the CHA program [12].

We conducted a complete mapping of the county and its communities prior to the first survey and then updated the mapping in partnership with the county government before each survey round to ensure complete capture of all communities and eligible participants First stage sampling (communities) was conducted with sampling probabilities proportional to size (PPS) in all three surveys. We explicitly stratified by intervention status in the 2015 survey but not in the subsequent surveys. However, we implicitly stratified by district and community size in all the surveys, and implicit stratification by district had the effect of also implicitly stratifying by implementation phase (since implementation was by district). Second stage sampling was by a modified random walk procedure in which we spun a triangle in the center of each community, used a random number generator to choose a first house between the center and margin of the community in the direction of each point, and then proceeded to the next nearest house in each direction.

We designed our sample to be able to measure all key indicators (child health treatment, maternal service utilization, and vaccine coverage) within a maximum of a 10-percentage point margin of error (with vaccine coverage usually being the least precisely estimated). We also planned for 80% power to detect a 10-percentage point increase in childhood illness treatment and facility delivery from baseline. Design effects were estimated based on DHS survey values at baseline and then updated for each survey based on the corresponding value for each indicator at the prior survey. We assumed design effects ranging from approximately 1.4 to 2.5 depending on the indicator and survey.

### Outcomes

Completion of the full maternal CoC was defined as receiving at least four antenatal care visits at home or in a health facility (the WHO standard during most of the 2-year recall periods in all 3 surveys) [34], and giving birth within a healthcare facility, and receiving effective early postnatal care coverage for the mother (provided by a trained clinician and occurring within 48 h of birth, either at a facility or at home). Completion of no parts of the continuum of care was defined as not meeting each of the above criteria for full maternal CoC, i.e., did not receive four ANC visits, and did not give birth within a healthcare facility, and did not receive effective early PNC coverage. Completion of each potential combination of ANC, FBD, and PNC were calculated to evaluate patterns in uptake of portions of the continuum of care. The survey only asked about completion of one maternal PNC visit, so completion of full WHO recommendations on PNC was not possible. Due to limitations in survey questions regarding content of care at ANC and PNC visits, our outcomes focused on contact with care only.

### Covariates

Based on previous literature, we included covariates that could impact the relationship between a CHA program and the maternal continuum of care [7–11, 13–16]. Community-level characteristics included distance to a health facility (from county-level mapping data determining each community’s road distance from the nearest health facility, split into those greater than 5 but less than 10 km from a facility, those at least 10 km but less than 20 km from a facility, and greater than 20 km from a facility), accessibility by motorbike, (given the additional travel barriers faced when accessing health care facilities for communities only accessible by walking) and whether the community was a mining community (given the socioeconomic and geographic differences for these communities) [35]. The household-level characteristics included household wealth, utilizing a metric calculated with an adjusted Demographic and Health Survey principal component analysis model derived from survey questions elucidating ownership of animals and household items as well as composition of house and hygiene features (split into quintiles) [36]. Maternal characteristics included maternal age (split into 18–29, 30–39, and 40 or greater), maternal education (no education vs. primary education or greater), language of survey completion (English, Bassa, or other), whether the mother had any previous children, how many children, whether any of her previous children had died (only available in 2021), and the gender of the most recent child born alive prior to the last birth. Maternal education, previous live births, and child gender were not captured in 2015 and thus no comparisons available against other timepoints for those variables. Missingness for all other data was no more than 2.3% for any variable.

### Analysis

Tests of differences in proportions were used to identify any significant changes in rates of continuum of care completion following CHA program implementation. Sensitivity analyses accounting for 2015 survey data impacted by disruptions to health care delivery during the 2014 Ebola epidemic in Liberia were also performed (although the epidemic continued to 2016 in Liberia, Rivercess only experienced cases in 2014). We removed births that occurred in the immediate 12 months prior to survey completion (for 2015 survey: June 2014 – May 2015) that captured the majority of the EVD outbreak in Liberia and the entirety of the EVD outbreak in Rivercess County specifically. This methodology was then carried over to the other two surveys (for 2018 survey: removing July 2017 – June 2018; for 2021 survey: removing December 2020 – November 2021). Multivariable logistic regression models were fitted to analyze the covariates that were associated with completion of all steps or no steps in the maternal continuum of care at baseline (2015 timepoint) and at follow up (combined 2018 and 2021 timepoints). All analyses were completed in Stata version 17.0 and sampling weights and standard errors were adjusted for the two stage cluster-sampling study design. We reported using the Strengthening the Reporting of Observational Studies in Epidemiology (STROBE) cross sectional version. (see Additional File 4).

## Results

### Descriptives

In Rivercess County, three surveys (2015, n = 354 women, from a total of n = 675 households originally surveyed; 2018, n = 312 women, n = 610 households surveyed; 2021, n = 302 women, n = 704 households surveyed) were analyzed following confirmation of eligibility criteria (Table 1). At baseline, the response rate was 94% for households and 98% among women in selected households for a composite response rate of 92%. In the 2018 survey, response rate for households was 98% and response rate for women was 92% for a composite response rate of 90%. In the 2021 survey, response rate for households was 77% and response rate for women was 96% for a composite response rate of 74%. Compared to baseline in 2015, participants in 2018 reported higher proportion of younger mothers, lower rates of living in a motorbike accessible community, and an increased mean wealth index for the lowest wealth quintile. Compared to baseline, participants in 2021 reported lower rates of living in a mining community, and an increased mean wealth index for the lowest wealth quintile. Participants in the follow up survey of 2018 differed from those in 2021 in their mean wealth index for the middle quintile and number of children, as well as in rates of no previous live births, education level, and living in motorbike accessible communities.

**Table 1:** Descriptive characteristics for responses from each survey

Category			2015 survey	2018 survey	2021 survey	***P***-values, 2015 survey vs. 2018 survey	***P***-values, 2018 survey vs. 2021 survey	***P***-values, 2015 survey vs. 2021 survey
	Number of households		309	280	283	-	-	-
	Number of women		354	312	302	-	-	-
			% (95% CI)/mean (95% CI)					
Community	Mining community		24.3(15.2, 36.5)	21.3 (10.7, 38.0)	10.9 (5.56 20.4)	0.74	0.19	**0.04**
	Motorbike acc. community		79.9 (68.5, 87.9)	59.9 (45.3, 73.0)	83.4 (72.6, 90.5)	**0.02**	**< 0.01**	0.60
	Distance to facility	≥5-<10 km	50.2 (38.9, 61.5)	46.8 (32.9, 61.2)	57.3 (43.2, 70.3)	0.18	0.51	0.56
		≥10-<20 km	45.6 (34.5, 57.2)	39.7 (26.4, 54.8)	36.1 (24.3, 49.9)			
		≥20 km	4.2 (1.7, 9.7)	13.5 (5.9, 27.7)	6.6 (1.5, 24.9)			
Household	Household wealth*	lowest quintile	-2.08 (-2.13, -2.03)	-1.83 (-1.91, -1.76)	-1.94 (-2.07. -1.82)	**< 0.01**	0.13	**0.048**
		2nd quintile	-1.23 (-1.27, -1.19)	-1.21 (-1.25, -1.17)	-1.22 (-1.29, -1.15)	0.57	0.82	0.85
		middle quintile	-0.60 (-0.70, -0.50)	-0.66 (-0.71, -0.61)	-0.55 (-0.60, -0.50)	0.29	**< 0.01**	0.37
		4th quintile	0.19 (0.08, 0.31)	0.29 (0.21, 0.37)	0.32 (0.25, 0.39)	0.17	0.58	0.06
		highest quintile	3.23 (2.40, 4.06)	3.42 (2.71, 4.13)	3.17 (2.65, 3.68)	0.71	0.55	0.90
Maternal	Maternal age	18–29	65.2 (60.7, 69.5)	60.6 (55.8, 65.2)	62.9 (58.3, 67.3)	**0.04**	0.67	0.21
		30–39	30.4 (26.2, 34.9)	30.8 (26.6, 35.3)	29.8 (25.11, 34.96)			
		40+	4.4 (2.7, 7.0)	8.7 (6.6, 11.3)	7.28 (5.2, 10.1)			
	Maternal education	No education	-	47.1 (40.7, 53.7)	33.8 (28.3, 39.7)	-	**< 0.01**	-
		Some primary education or more	-	52.9 (46.3, 59.4)	66.2 (60.3, 71.7)			
	Maternal survey language	English	38.5 (31.1, 46.4)	49.0 (39.2, 59.0)	38.4 (30.3, 47.2)	0.11	0.15	0.54
		Bassa	61.5 (53.6, 68.9)	50.6 (40.8, 60.4)	61.3 (52.4, 69.4)			
		Other	0	0.3 (0.1, 1.9)	0.3 (0.1, 2.0)			
	Number of children born alive		-	4.0 (3.7, 4.3)	3.2 (3.0, 3.5)	-	**< 0.01**	-
	Any children passed		-	-	11.9 (8.9, 15.8)	-	-	-
Birth	No previous live births		-	16.7 (12.9, 21.3)	22.9 (18.7, 27.6)	-	**0.046**	-
	Female child		-	53.6 (49.4, 57.7)	48.2 (42.4, 54.0)	-	0.63	-
	Antenatal care (any)		91.7 (87.4, 94.6)	97.1 (94.6, 98.5)	98.3 (95.5, 99.4)	**< 0.01**	0.34	**< 0.01**
	≥ 4 antenatal care visits		67.8 (61.2, 73.7)	79.6 (73.0, 84.9)	82.1 (76.3, 86.6)	**< 0.01**	0.54	**< 0.01**
	Facility-based delivery		56.3 (48.9, 63.3)	75.6 (68.4, 81.6)	92.7 (89.2, 95.2)	**< 0.01**	**< 0.01**	**< 0.01**
	Postnatal care (any)		63.1 (57.8, 68.1)	84.9 (80.6, 88.4)	81.1 (76.2, 85.2)	**< 0.01**	0.21	**< 0.01**
	Postnatal care (effective)		37.2 (31.6, 43.1)	75.0 (69.7, 79.6)	72.2 (66.5, 77.2)	**< 0.01**	0.45	**< 0.01**

All results are weighted, and percentages and means are reported with 95% confidence intervals. P values for equivalence testing across years are also reported, with significant differences bolded. Results with dashes indicate results that could not be computed due to missing survey questions from 2015

*Household wealth score as measured in a principle component analysis based on DHS scoring methodology

### Continuum of care completion rates

We saw an increase in all three steps of the maternal continuum of care following implementation of the CHA program. Percentage of births including at least four antenatal care visits increased from 67.8% (weighted 95% confidence interval (CI) 61.2, 73.7) in 2015 to 79.6% (95% CI 73.0, 84.9) in 2018 and 82.1% (95% CI 76.3, 86.6) in 2021. The percentage of births taking place in a health care facility in 2015 was 56.3% (95% CI 48.9, 63.3), increasing to 75.6% (95% CI 68.4, 81.6) in 2018 and even further rising to 92.7% (95% CI 89.2, 95.2) in 2021. Percentage of women receiving postnatal care from a formal provider and within 48 h of birth rose from 37.2% (95% CI 31.6, 43.1) at baseline to 75.0% (95% CI 69.7, 79.6) in 2018 and 72.2% (95% CI 66.5, 77.2) in 2021.

**Fig. 1:**
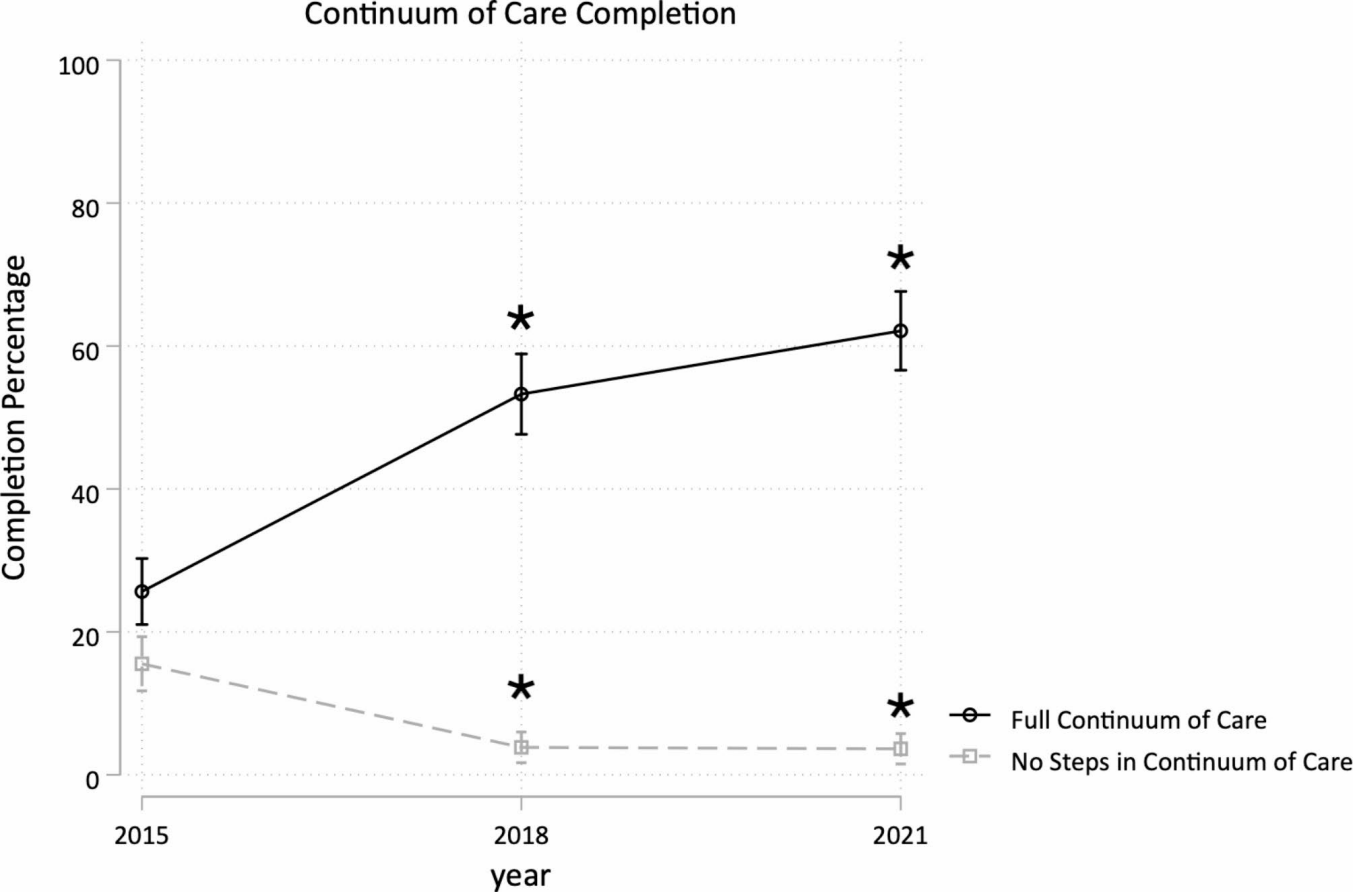
Change in continuum of care rates over time. Asterisks indicate significant change (p < 0.05) from pre-CHA implementation in 2015

**Fig. 2:**
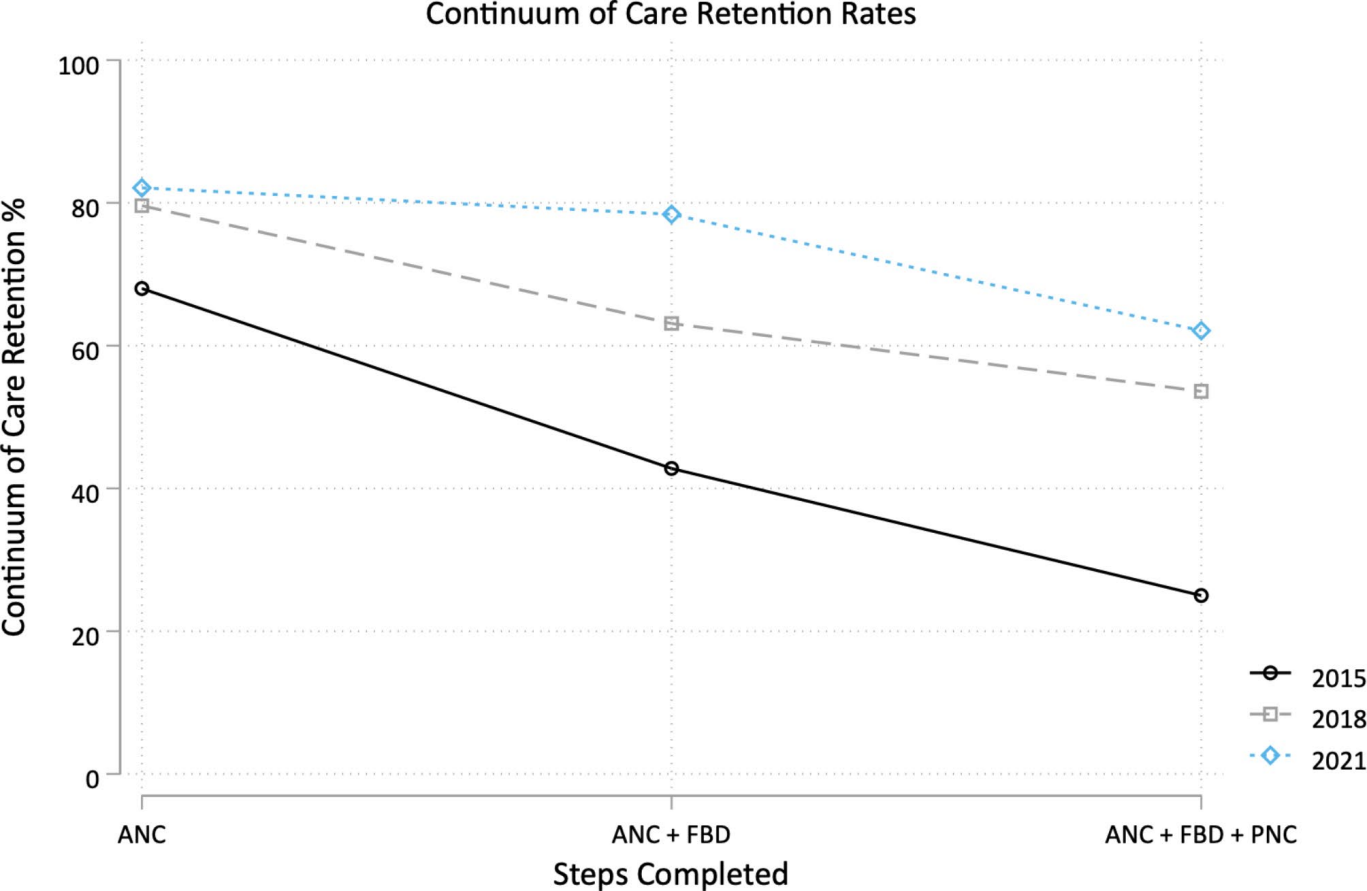
Continuum care retention rates over time. Retention in the continuum of care (across antenatal care, facility-based delivery, and postnatal care) tracked for each survey year

Changes in maternal continuum of care completion over all three surveys are illustrated in Fig. 1; Table 2. Increases in completion rates were observed after implementation of the CHA program from baseline to 2018 (29.7% points, 95% confidence interval (CI) [21.0, 38.4]), with maintenance but not further statistically significant increase from 2018 to 2021 (8.9pp [95% CI -0.1, 18.0]). Following CHA program implementation, the percentage of respondents who completed no steps of the continuum of care also decreased from 17.6% in 2015 to 4.0% (-12.4pp [95% CI -17.6, -7.2]) in 2018 and 3.7% (-12.6pp [95% CI -17.5, -7.7]) in 2021. Full results for percentages of respondents per possible combination of continuum of care step completion are described in the appendix (Additional File 5), with highlights of retention patterns over time illustrated in Fig. 2.

**Table 2:** Change in continuum of care rates over time

	2015	2018	2021	2015–2018 change (95% CI)	***P***	2018–2021 change	***P***	2015–2021 change	***P***
Full maternal continuum of care	23.6%	53.4%	62.1%	**29.7pp (21.0, 38.4)**	**< 0.001**	8.9pp (-0.1, 18.0)	0.06	**38.6pp (30.4, 46.7)**	**< 0.001**
No steps in the maternal continuum of care	16.2%	3.9%	3.6%	**-12.4pp (-17.6, -7.1)**	**< 0.001**	-0.2pp (-0.4, 0.03)	0.91	**-12.6pp (-17.5, -7.7)**	**< 0.001**

Bolded results indicate significant change in continuum of care rate in percentage points (pp) over time

### Sensitivity analyses adjusting for ebola epidemic

In sensitivity analyses, significant increases in full continuum of care completion and significant decreases in completion of no continuum of care steps after CHA implementation were still observed – see appendix (Additional File 6).

### Factors associated with continuum of care rates

In multivariate regression models, living between 10 and 20 km from a health facility was negatively associated (adjusted odds ratio (aOR) = 0.38, 95% confidence interval= [0.20, 0.71], p = < 0.01) with completing the full continuum of care (i.e., completion of all steps) before implementation of the National Community Health Assistant Program. Additionally, increased wealth was positively associated (middle wealth quintile: aOR = 2.32 [1.05, 5.15], p = 0.04; 4th wealth quintile: aOR = 2.72 [1.13, 6.58], p = 0.03; highest wealth quintile: aOR = 2.29 [1.09, 4.81], p = 0.03) with completing the full maternal continuum of care prior to implementation. In follow up years, there was no longer a significant association between wealth and full continuum of care completion, though living between 10 and 20 km from a health facility maintained a negative association with the full CoC (aOR = 0.54 [0.32, 0.92], p = 0.02). Additionally, living in a motorbike accessible community emerged as positively associated with completing the full CoC following implementation (aOR = 1.85 [1.11, 3.06], p = 0.02).

Prior to CHA program implementation, increased distance from health care facilities was positively associated with completing no steps in the continuum of care (10–20 km away: aOR = 1.94 [1.01, 3.71], p = 0.046; ≥20 km away: aOR = 3.09 [1.19, 8.02], p = 0.02) as compared to those residing between 5 and 10 km away. Increased maternal age (aOR = 2.91 [1.10, 7.73], p = 0.03) was also positively associated with no CoC. Following implementation, increased distance maintained a positive association with absence of maternal care 10–20 km away: aOR = 11.97 [2.75, 52.16], p < 0.01; ≥20 km away: aOR = 7.72 [1.46, 40.95], p = 0.02), while living in a mining community emerged as negatively associated with completing no CoC steps (aOR = 0.18 [0.04, 0.96], p = 0.045) (Table 3).

**Table 3:** Factors associated with completion of all steps or no steps in the maternal continuum of care (CoC).

Covariate		Full CoC				No CoC			
		Baseline		Follow up		Baseline		Follow up	
		aOR	P	aOR	***P***	aOR	***P***	aOR	***P***
Mining community		0.75 (0.37, 1.52)	0.42	0.94 (0.56, 1.58)	0.82	1.52 (0.72, 3.20)	0.26	**0.18 (0.04, 0.96)**	**0.045**
Motorbike acc. community		0.95 (0.48, 1.89)	0.88	**1.85 (1.11, 3.06)**	**0.02**	0.51 (0.26, 1.01)	0.06	1.45 (0.50, 4.22)	0.49
Distance to facility (ref: ≥5-<10 km)	≥10-<20 km	**0.38 (0.20, 0.71)**	**< 0.01**	**0.54 (0.32, 0.92)**	**0.02**	**1.94 (1.01, 3.71)**	**0.046**	11.97 (2.75, 52.16)	**< 0.01**
	≥20 km	0.46 (0.12, 1.72)	0.24	0.58 (0.30, 1.13)	0.11	**3.09 (1.19, 8.02)**	**0.02**	**7.72 (1.46, 40.95)**	**0.02**
Household wealth PCA quintile (ref: lowest quintile)	2nd	1.41 (0.61, 3.25)	0.41	1.44 (0.69, 3.00)	0.32	0.72 (0.34, 1.54)	0.39	1.01 (0.18, 5.61)	0.99
	middle	**2.32 (1.05, 5.15)**	**0.04**	0.70 (0.37, 1.32)	0.27	1.64 (0.74, 3.63)	0.22	1.84 (0.31, 11.04)	0.50
	4th	**2.72 (1.13, 6.58)**	**0.03**	0.70 (0.35, 1.39)	0.31	0.61 (0.20, 1.82)	0.37	1.47 (0.37, 5.86)	0.58
	highest	**2.29 (1.09, 4.81)**	**0.03**	1.38 (0.72, 2.64)	0.33	0.10 (0.01, 1.10)	0.06	1.51 (0.32, 7.16)	0.60
Maternal age (ref: 18–29)	30–39	1.00 (0.58, 1.72)	0.99	1.57 (0.98, 2.51)	0.06	0.73 (0.37, 1.43)	0.35	0.93 (0.36, 2.37)	0.87
	40+	0.56 (0.19, 1.63)	0.28	1.38 (0.63, 3.01)	0.42	**2.91 (1.10, 7.73)**	**0.03**	0.96 (0.11, 8.33	0.97
Some maternal education (reference: no education)		-	-	0.87 (0.60, 1.25)	0.44	-	-	1.34 (0.35, 5.18)	0.67
Survey completed in Bassa (reference: English)		1.50 (0.88, 2.54)	0.13	1.03 (0.69, 1.53)	0.90	0.77 (0.38, 1.56)	0.46	2.41 0.93, 6.21)	0.07
No previous live births		-	-	1.04 (0.68, 1.58)	0.87	-	-	0.58 (0.21, 1.67)	0.31
Female child		-	-	1.32 (0.88, 1.97)	0.17	-	-	1.08 (0.50, 2.31)	0.85

Adjusted odds ratios (95% confidence intervals) and *p* values for multivariate logistic regression models are reported. Baseline (2015) and combined follow up (2018, 2021) are utilized. Significant results are bolded. Results with dashes indicate results that could not be computed due to missing survey questions from 2015

## Discussion

In this study, we found that implementation of the national CHA program in rural Liberia was associated with improvements in both completion of the full maternal continuum of care (reaching rates double that at baseline), as well as with reduction in women and their babies who had no formal care (reduced to less than 5% of births). This improvement persisted even during the COVID-19 pandemic, with the first cases reported in March 2020, and when many countries reported a decrease in important maternal and neonatal care. The results were also robust to sensitivity analyses accounting for potential artificial depression of baseline rates due to the 2014 Ebola epidemic.

Distance has been associated with decreased access to and uptake of important maternal and neonatal care in rural settings in countries in Africa [14, 37]. Pre-implementation, we also found that living between 10 and 20 km (but not 20 or more km) from a health facility was associated with poorer outcomes in completion of the maternal continuum of care. This may be attributed to the low sample size at baseline (4.2%) of respondents living 20 km or more from a facility, leading to the inability to detect significant associations. Following implementation, living between 10 and 20 km maintained association with non-completion of the full continuum of care, and both levels of far distance from a health facility were associated with receiving no steps in the care cascade. Additionally, living in a motorbike-accessible community was associated with increased completion of the full maternal CoC following implementation. Surprisingly, living in a mining community was associated with lower rates of no care following implementation. Previously, mining communities have been associated with smaller rates of increasing care following NCHAP implementation [25]. Our finding may be a result of mining communities often having higher rates of private methods of health care and pharmaceutical access, which may provide extra catchment alongside increased CHA presence to reduce the numbers of women who access no care across the maternal continuum [25].

These results demonstrate a continued need for focus on improving access to care for those with the greatest need, which is in accordance with NCHAP’s goal of explicitly reaching those living in communities far from a health facility. Additionally, this study’s data showed reduction of significant wealth disparities in completing the full CoC, but not avoiding no CoC completion, following CHA implementation. We hypothesize that baseline differences in CoC rates based on relative wealth differences disappeared after implementation do to the NCHAP equitably improving access by showing the most significant increases for those with the fewest resources. However, the lack of association between wealth status and poor CoC at baseline is surprising and may be an indicator that the relative household wealth index does not appreciably stratify a population that is almost uniformly underresourced when evaluating a rare outcome such as receiving no CoC. These results support previous literature indicating the baseline presence of disparities in continuum of care outcomes associated with differences in wealth and distance to health care facilities [7–13, 17], while strengthening the evidence that community-based interventions are important strategies for achieving equity [38].

The data from 2021 importantly demonstrated no decrease in reported maternal care during a time period that overlapped with the first 19 months of the COVID-19 pandemic (surveys in October/November 2021 included births from fall of 2019 to fall of 2021). This is in contrast to a report that select health care facilities in Liberia experienced a decline in ANC and FBD rates from March and April 2020 until December 2020 [39], as well as a global review illustrating declines in antenatal care and mixed reports of postnatal care rates during the pandemic [40]. Other African countries have witnessed various degrees of reductions of continuum of care service utilization and delayed or decreased care following introduction of COVID-19 restrictions [41, 42]. Hypotheses for why our study’s findings differ from these other studies include a focus on very remote communities that have experienced fewer COVID cases [43] while benefitting from an established, government-supported CHW program that has had many years to build trust and promote seeking care.

Our results contribute to existing knowledge of the value of CHW programs but specifically focus on the association of CHWs with utilization throughout the spectrum of maternal and early neonatal care continuum. These results report specific outcomes related to one county in Liberia but are similar to global review findings that CHW program implementations are successful at improving access to care at each individual stage in the continuum of care [19, 20]. Expanding the reach of these community-based interventions are key to reducing neonatal and infant mortality rates, as evidenced by one study finding that reaching 90% coverage of a set of CHW interventions is estimated to reduce under-five mortality by 35% over five years [44]. Liberia is actively working to achieve these goals, as the NCHAP has since expanded to every Liberian county, reaching approximately four out of five people living over five km from the nearest health facility [24].

Our study has a number of limitations. We used retrospective self-reported uptake of the steps along the continuum of care, which could introduce opportunities for recall bias or overreporting of health care use due to social desirability. The sample sizes of each survey prevented our analyses from examining association of use of the continuum of care on infant and child mortality rates. Survey content inconsistencies resulted in missing 2015 maternal education and birth characteristics. Because implementation covered the full county simultaneously, no comparison group was available—though the large effect size observed in this population is unlikely without other simultaneous large-scale health interventions, which we know were not present. Finally, we did not include qualitative data collection to understand the barriers and facilitators to completion of steps and the full continuum of care. Additional studies with enough power to examine impacts on infant and child mortality rates as well as completion of essential newborn care are warranted for understanding how maternal counseling by CHAs can influence neonatal outcomes.

## Conclusions

In conclusion, we found that implementation of the national CHA program was associated with a sustained increased in completion of maternal continuum of care and importantly, associated with a reduction in mothers receiving none of the maternal continuum of care steps as well as reduction in wealth as a factor associated with gaps in care, though there remains a demonstrated need to continue to improve rates for those living farthest from health facilities. This evidence points toward the capacity for CHW programs in rural areas to equitably drive participation in maternal and neonatal care that could contribute to reducing maternal and neonatal mortality. Community-based interventions focused on increasing delivery of essential care to rural areas can succeed and can continue to be improved to fully reach all those in need.

### Electronic supplementary material

Below is the link to the electronic supplementary material.

Supplementary Material 1

Supplementary Material 2

Supplementary Material 3

Supplementary Material 4

Supplementary Material 5

Supplementary Material 6

## Data Availability

Deidentified datasets can be made available upon request to the corresponding author SDB.

## Abbreviations

ANC: Antenatal Care
CHA: Community Health Assistant
CoC: Continuum of Care
FBD: Facility–based Delivery
NCHAP: National Community Health Assistant Program
PNC: Postnatal Care

## References

[1] WHO. Fact Sheet: Maternal Mortality: World Health Organization. 2023 [Available from: https://www.who.int/news-room/fact-sheets/detail/maternal-mortality.

[2] WHO (2022). WHO recommendations on postnatal care of the Mother and Newborn.

[3] WHO. WHO recommendations on antenatal care for a positive pregnancy experience. 2018. 28079998

[4] WHO. WHO recommendations. Intrapartum care for a positive childbirth experience. 2018. 30070803

[5] Benova L, Owolabi O, Radovich E, Wong KLM, Macleod D, Langlois EV (2019). Provision of postpartum care to women giving birth in health facilities in sub-saharan Africa: a cross-sectional study using demographic and Health Survey data from 33 countries. PLoS Med.

[6] Doctor HV, Radovich E, Benova L (2019). Time trends in facility-based and private-sector Childbirth care: analysis of demographic and health surveys from 25 sub-saharan African countries from 2000 to 2016. J Glob Health.

[7] Aryastami NK, Mubasyiroh R. Traditional practices influencing the use of maternal health care services in Indonesia. PLoS ONE. 2021;16(9). 10.1371/journal.pone.0257032 PMC8432883 34506525

[8] Chham S, Radovich E, Buffel V, Ir P, Wouters E. Determinants of the continuum of maternal health care in Cambodia: an analysis of the Cambodia demographic health survey 2014. BMC Pregnancy Childbirth. 2021;21(1). 10.1186/s12884-021-03890-7 PMC8170811 34078318

[9] Fekadu GA, Ambaw F, Kidanie SA. Facility delivery and postnatal care services use among mothers who attended four or more antenatal care visits in Ethiopia: further analysis of the 2016 demographic and health survey. BMC Pregnancy Childbirth. 2019;19(1). 10.1186/s12884-019-2216-8 PMC6371418 30744583

[10] Gandhi S, Gandhi S, Dash U, Suresh Babu M. Predictors of the utilisation of continuum of maternal health care services in India. BMC Health Serv Res. 2022;22(1). 10.1186/s12913-022-07876-9 PMC9069727 35513830

[11] Khatri RB, Karkee R, Durham J, Assefa Y. Universal coverage of the first antenatal care visit but poor continuity of care across the maternal and newborn health continuum among Nepalese women: analysis of levels and correlates. Globalization and Health. 2021;17(1). 10.1186/s12992-021-00791-4 PMC8665493 34895276

[12] Moran AC, Kerber K, Sitrin D, Guenther T, Morrissey CS, Newby H et al. Measuring Coverage in MNCH: indicators for Global Tracking of Newborn Care. PLoS Med. 2013;10(5). 10.1371/journal.pmed.1001415 PMC3646209 23667335

[13] Wang W, Hong R. Levels and determinants of continuum of care for maternal and newborn health in Cambodia-evidence from a population-based survey. BMC Pregnancy Childbirth. 2015;15(1). 10.1186/s12884-015-0497-0 PMC4371879 25885596

[14] Alem AZ, Shitu K, Alamneh TS. Coverage and factors associated with completion of continuum of care for maternal health in sub-saharan Africa: a multicountry analysis. BMC Pregnancy Childbirth. 2022;22(1). 10.1186/s12884-022-04757-1 PMC9121540 35590260

[15] Emiru AA, Alene GD, Debelew GT. Women’s retention on the continuum of maternal care pathway in west Gojjam zone, Ethiopia: Multilevel analysis. BMC Pregnancy Childbirth. 2020;20(1). 10.1186/s12884-020-02953-5 PMC7191802 32349687

[16] Mohan D, LeFevre AE, George A, Mpembeni R, Bazant E, Rusibamayila N (2017). Analysis of dropout across the continuum of maternal health care in Tanzania: findings from a cross-sectional household survey. Health Policy Plann.

[17] Kenny A, Basu G, Ballard M, Griffiths T, Kentoffio K, Niyonzima JB et al. Remoteness and maternal and child health service utilization in rural Liberia: a population-based survey. J Global Health. 2015;5(2). 10.7189/jogh.05.020401 PMC4512264 26207180

[18] Gogia S, Sachdev HS (2010). Home visits by community health workers to prevent neonatal deaths in developing countries: a systematic review. Bull World Health Organ.

[19] Jennings MC, Pradhan S, Schleiff M, Sacks E, Freeman PA, Gupta S et al. A comprehensive review of the evidence regarding the effectiveness of community-based primary health care in improving maternal, neonatal and child health: 2. Maternal health findings. J Global Health. 2017;7(1). 10.7189/jogh.07.010902 PMC5491947 28685040

[20] Sacks E, Freeman PA, Sakyi K, Jennings MC, Rassekh BM, Gupta S et al. Comprehensive review of the evidence regarding the effectiveness of community-based primary health care in improving maternal, neonatal and child health: 3. Neonatal health findings. J Global Health. 2017;7(1). 10.7189/jogh.07.010903 PMC5491944 28685041

[21] Agarwal S, Curtis S, Angeles G, Speizer I, Singh K, Thomas J. Are community health workers effective in retaining women in the maternity care continuum? Evidence from India. BMJ Global Health. 2019;4(4). 10.1136/bmjgh-2019-001557 PMC6666803 31406590

[22] Gilmore B, McAuliffe E. Effectiveness of community health workers delivering preventive interventions for maternal and child health in low- and middle-income countries: a systematic review. BMC Public Health. 2013. 10.1186/1471-2458-13-847 PMC3848754 24034792

[23] Lewin S, Munabi-Babigumira S, Glenton C, Daniels K, Bosch-Capblanch X, van Wyk BE (2010). Lay health workers in primary and community health care for maternal and child health and the management of infectious Diseases.

[24] Healey J, Wiah SO, Horace JM, Majekodunmi DB, Duokie DS. Liberia’s Community Health Assistant Program: Scale, Quality, and Resilience. 2020. 10.9745/GHSP-D-20-00509 PMC7971381 33727317

[25] Luckow PW, Kenny A, White E, Ballard M, Dorr L, Erlandson K (2017). Implementation research on community health workers’ provision of maternal and child health services in rural Liberia. Bull World Health Organ.

[26] Chen N, Dahn B, López Castañeda C, Muther K, Panjabi R, Price M et al. Community Health Workers in Liberia. Exemplars in Global Health. 2019.

[27] White EE, Downey J, Sathananthan V, Kanjee Z, Kenny A, Waters A (2018). A community health worker intervention to increase Childhood Disease treatment coverage in rural Liberia: A controlled before-and-after evaluation. Am J Public Health.

[28] Program TD. Liberia Demographic and Health Survey 2013. 2013.

[29] Program TD. Liberia 2019-20 Demographic and Health Survey Summary Report. Demographic and Health Program; 2021.

[30] Shapira G, Ahmed T, Drouard SHP, Amor Fernandez P, Kandpal E, Nzelu C (2021). Disruptions in maternal and child health service utilization during COVID-19: analysis from eight sub-saharan African countries. Health Policy Plan.

[31] Liberia Institute of S, Geo-Information S. Republic of Liberia 2008 National Population and Housing Census Final Results. 2009.

[32] Liberia Institute of S, Geo-Information Services MoH., Icf. Liberia Demographic and Health Survey 2019-20. Monrovia, Liberia and Rockville, Maryland, USA; 2021.

[33] Ly J, Sathananthan V, Griffiths T, Kanjee Z, Kenny A, Gordon N (2016). Facility-based delivery during the Ebola Virus Disease Epidemic in Rural Liberia: analysis from a cross-sectional, Population-based Household Survey. PLoS Med.

[34] WHO. WHO recommendations on antenatal care for a positive pregancy experience. 2016. 28079998

[35] Thaddeus S, Maine D. Too Far to Walk: Maternal Mortality in Context. Newsl Womens Glob Netw Reprod Rights. 1991;(36). 12284530

[36] Filmer D, Pritchett LH. Estimating Wealth Effects without Expenditure Data-or Tears: An Application to Educational Enrollments in States of India. 2001. 10.1353/dem.2001.0003 11227840

[37] Tlebere P, Jackson D, Loveday M, Matizirofa L, Mbombo N, Doherty T (2007). Community-based situation analysis of maternal and neonatal care in South Africa to explore factors that impact utilization of maternal health services. J Midwifery Womens Health.

[38] Schleiff M, Kumapley R, Freeman PA, Gupta S, Rassekh BM, Perry HB. Comprehensive review of the evidence regarding the effectiveness of community-based primary health care in improving maternal, neonatal and child health: 5. Equity effects for neonates and children. J Global Health. 2017;7(1). 10.7189/jogh.07.010905 PMC5491949 28685043

[39] Aranda Z, Binde T, Tashman K, Tadikonda A, Mawindo B, Maweu D (2022). Disruptions in maternal health service use during the COVID-19 pandemic in 2020: experiences from 37 health facilities in low-income and middle-income countries.

[40] Townsend R, Chmielewska B, Barratt I, Kalafat E, van der Meulen J, Gurol-Urganci I (2021). Global changes in maternity care provision during the COVID-19 pandemic: a systematic review and meta-analysis. EClinicalMedicine.

[41] Adu PA, Stallwood L, Adebola SO, Abah T, Okpani AI (2022). The direct and indirect impact of COVID-19 pandemic on maternal and child health services in Africa: a scoping review. Glob Health Res Policy.

[42] Ameyaw EK, Ahinkorah BO, Seidu AA, Njue C (2021). Impact of COVID-19 on maternal healthcare in Africa and the way forward. Arch Public Health.

[43] Akpan GE, Bawo L, Amo-Addae M, Kennedy J, Wesseh CS, Whesseh F (2022). COVID-19 reinfection in Liberia: implication for improving Disease surveillance. PLoS ONE.

[44] Chou VB, Friberg IK, Christian M, Walker N, Perry HB (2017). Expanding the population coverage of evidence-based interventions with community health workers to save the lives of mothers and children: an analysis of potential global impact using the lives Saved Tool (LiST). J Glob Health.

