# Supplementary material for: A repeated cross-sectional study of the association of community health worker intervention with the maternal continuum of care in rural Liberian communities
Source: BMC Pregnancy Childbirth. 2023 Dec 7;23:841. doi: 10.1186/s12884-023-06162-8 (PMC10701987; doi:10.1186/s12884-023-06162-8)
Supplement: Supplementary file 4 — Supplementary Material 4 [file 12884_2023_6162_MOESM4_ESM.docx]

Additional File 4: Completed STROBE checklist

|  | Item No. | Recommendation | Page  No. | Relevant text from manuscript |
| --- | --- | --- | --- | --- |
| **Title and abstract** | 1 | (*a*) Indicate the study’s design with a commonly used term in the title or the abstract | 1 | A repeated cross-sectional study of community health worker intervention in association with the maternal continuum of care in rural Liberian communities. |
|  |  | (*b*) Provide in the abstract an informative and balanced summary of what was done and what was found | 3-4 | We analyzed repeated cross-sectional household surveys of women of childbearing age living at least five kilometers from a health care facility in Rivercess County, Liberia. We measured the survey-weighted, before-to-after implementation difference in completion of all steps and no steps in the maternal continuum of care. We used multivariable regression to explore covariates associated with completion rates before and after NCHAP implementation.  Following implementation of the NCHAP, the completion rate of the full maternal continuum of care in Rivercess County more than doubled while the rate of completing no steps in the continuum fell below 5%. These rates were sustained over time with reduced differences across wealth groups, although very far distance remained a risk for no steps. |
| Introduction | | | |  |
| Background/rationale | 2 | Explain the scientific background and rationale for the investigation being reported | 5-6 | Ensuring uptake of evidence-based care throughout pregnancy, delivery and post-partum periods is critical to the health of mothers and their neonates (1). Receipt of these components of health delivery across the maternal continuum of care (CoC) has been associated with reductions in maternal and neonatal morbidity and mortality (2). Updated recommendations include eight antenatal contacts, delivery by trained birth attendants in a hygienic and adequately-supplied facility, and four postnatal contacts (3, 4). Despite these recommendations, access to and completion of the full maternal continuum of care remains limited, especially for low- and middle- income countries in Sub-Saharan Africa (5, 6).  Community health worker (CHW) interventions have been found to contribute to increased rates in various steps of the maternal continuum of care individually … CHWs were also associated with increased rates of antenatal care and presence of skilled birth attendants, but not completion of the full continuum of care (21). However, fewer studies examined rates of postnatal care access, and have called for more community-based interventions in Africa to match the success of similar programs implemented in regions such as south Asia(20). |
| Objectives | 3 | State specific objectives, including any prespecified hypotheses | 7 | In this study, we examined if implementation of the National CHA Program was associated with increased completion of the maternal continuum of care in Rivercess County. We also explored factors associated with retention of women at steps in the maternal continuum of care from antenatal through postnatal care. |
| Methods | | | |  |
| Study design | 4 | Present key elements of study design early in the paper | 8 | The study utilized existing data collected over time from a repeated cross-sectional household survey of communities served by the CHA program. The survey was adapted from the Liberian Demographic and Health Survey (31). We included surveys completed before NCHAP implementation in 2015 and then post-implementation surveys in 2018 and 2021. |
| Setting | 5 | Describe the setting, locations, and relevant dates, including periods of recruitment, exposure, follow-up, and data collection | 8 | The study was completed in Rivercess County, in which the National CHA Program implementation is supported by Last Mile Health. Rivercess is a majority-rural county located along Liberia’s central coast with population estimate of 71,000, divided into six health districts (29). The CHA program in Rivercess County was implemented simultaneously across the districts in August of 2015. Implementation included recruitment, training, and deployment of CHAs back into their communities utilizing a 5 S’s approach: selection, skills, supervision, salary, and supplies. CHAs are trained in facilitating access to and receipt of maternal, and newborn health with full details in Luckow, et al. (25, 30). |
| Participants | 6 | (*a*) *Cohort study*—Give the eligibility criteria, and the sources and methods of selection of participants. Describe methods of follow-up  *Case-control study*—Give the eligibility criteria, and the sources and methods of case ascertainment and control selection. Give the rationale for the choice of cases and controls  *Cross-sectional study*—Give the eligibility criteria, and the sources and methods of selection of participants | 8 | Women who completed the survey between the ages of 18 and 49 who lived in a community at least five kilometers from the nearest health care facility and who reported a birth within the last two years of the time surveyed were included. |
|  |  | (*b*) *Cohort study*—For matched studies, give matching criteria and number of exposed and unexposed  *Case-control study*—For matched studies, give matching criteria and the number of controls per case | N/A | N/A |
| Variables | 7 | Clearly define all outcomes, exposures, predictors, potential confounders, and effect modifiers. Give diagnostic criteria, if applicable | 9-10 | Completion of the full maternal continuum of care was defined as receiving at least four antenatal care visits (the WHO standard during the survey periods), giving birth within a healthcare facility, and receiving effective early postnatal care coverage (provided by a trained clinician and occurring within 48 hours of birth).  Community-level characteristics included distance to a health facility (from county-level mapping data determining each community’s distance from the nearest health facility, split into those greater than 5 but less than 10 kilometers from a facility, those at least 10 kilometers but less than 20 kilometers from a facility, and greater than 20 kilometers from a facility), accessibility by motorbike, (given the additional travel barriers faced when accessing health care facilities for communities only accessible by walking) and whether the community was a mining community (given the socioeconomic and geographic differences for these communities) (33). The household-level characteristic included household wealth, utilizing a metric calculated with an adjusted Demographic and Health Survey principal component analysis model derived from survey questions elucidating ownership of animals and household items as well as composition of house and hygiene features (split into quintiles) (34). Maternal characteristics included maternal age (split into 18-29, 30-39, and 40 or greater), maternal education (no education vs. primary education or greater), language of survey completion (English, Bassa, or other), whether she had any previous children, whether any children had previously died (only available in 2021), and child gender of most recent birth. |
| Data sources/ measurement | 8* | For each variable of interest, give sources of data and details of methods of assessment (measurement). Describe comparability of assessment methods if there is more than one group | 9-10 | See above for sources and comparability methods |
| Bias | 9 | Describe any efforts to address potential sources of bias |  |  |
| Study size | 10 | Explain how the study size was arrived at | 8-10 | The household survey methods are described in detail elsewhere (32). Briefly, households in communities located five or more kilometers from the nearest primary care health center were selected at random through a population-representative two-stage cluster-sample that first randomly selected communities and then households within selected communities. …  Women who completed the survey between the ages of 18 and 49 who lived in a community at least five kilometers from the nearest health care facility and who reported a birth within the last two years of the time surveyed were included. The 2021 survey included 15-17-year-olds, but they were excluded to maintain consistency with the previous surveys. |

Continued on next page

| Quantitative variables | 11 | Explain how quantitative variables were handled in the analyses. If applicable, describe which groupings were chosen and why | 9-10 | See above for descriptions of all variables and their use |
| --- | --- | --- | --- | --- |
| Statistical methods | 12 | (*a*) Describe all statistical methods, including those used to control for confounding | 10 | Tests of differences in proportions were used to identify any significant changes in rates of continuum of care completion following CHA program implementation. … Multivariable logistic regression models were fitted to analyze the covariates that were associated with completion of all steps or no steps in the maternal continuum of care. |
|  |  | (*b*) Describe any methods used to examine subgroups and interactions | N/A | N/A |
|  |  | (*c*) Explain how missing data were addressed | 10 | Maternal education, number of children, and child gender were not captured in 2015 and thus no comparisons available against other timepoints for those variables. Missingness for all other data was no more than 2.3% for any variable. |
|  |  | (*d*) *Cohort study*—If applicable, explain how loss to follow-up was addressed  *Case-control study*—If applicable, explain how matching of cases and controls was addressed  *Cross-sectional study*—If applicable, describe analytical methods taking account of sampling strategy | 10 | Sampling weights and standard errors were adjusted for clustering. |
|  |  | (*e*) Describe any sensitivity analyses | 10 | Sensitivity analyses accounting for data impacted by disruptions to health care delivery during the 2014 Ebola epidemic in Liberia were also performed |
| Results | | | | |
| Participants | 13* | (a) Report numbers of individuals at each stage of study—eg numbers potentially eligible, examined for eligibility, confirmed eligible, included in the study, completing follow-up, and analysed | 10 | In Rivercess County, three timepoints (2015, n = 354; 2018, n = 312; 2021, n = 302) were analyzed following confirmation of eligibility criteria |
|  |  | (b) Give reasons for non-participation at each stage | 9 | Women who completed the survey between the ages of 18 and 49 who lived in a community at least five kilometers from the nearest health care facility and who reported a birth within the last two years of the time surveyed were included. |
|  |  | (c) Consider use of a flow diagram | N/A | N/A |
| Descriptive data | 14* | (a) Give characteristics of study participants (eg demographic, clinical, social) and information on exposures and potential confounders | 10-11 | Compared to baseline in 2015, participants in 2018 reported higher proportion of younger mothers, lower rates of living in a motorbike accessible community, and an increased mean wealth index. Compared to baseline, participants in 2021 reported lower rates of living in a mining community, and an increased mean wealth index. Follow up surveys of 2018 and 2021 differed in mean wealth index and number of children, as well as in rates of first birth, education level, and living in motorbike accessible communities. |
|  |  | (b) Indicate number of participants with missing data for each variable of interest | 10 | Maternal education, number of children, and child gender were not captured in 2015 and thus no comparisons available against other timepoints for those variables. Missingness for all other data was no more than 2.3% for any variable. |
|  |  | (c) *Cohort study*—Summarise follow-up time (eg, average and total amount) | N/A | N/A |
| Outcome data | 15* | *Cohort study*—Report numbers of outcome events or summary measures over time | N/A | N/A |
|  |  | *Case-control study—*Report numbers in each exposure category, or summary measures of exposure | N/A | N/A |
|  |  | *Cross-sectional study—*Report numbers of outcome events or summary measures | 11 | We saw an increase in all three steps of the maternal continuum of care following implementation of the CHA program. Percentage of births including at least four antenatal care visits increased from 67.8% (weighted 95% confidence interval (CI) 61.2, 73.7) in 2015 to 79.6% (95% CI 73.0, 84.9) in 2018 and 82.1% (95% CI 76.3, 86.6) in 2021. The percentage of births taking place in a health care facility in 2015 was 56.3% (95% CI 48.9, 63.3), increasing to 75.6% (95% CI 68.4, 81.6) in 2018 and even further rising to 92.7% (95% CI 89.2, 95.2) in 2021. Percentage of women receiving postnatal care from a formal provider and within 48 hours of birth rose from 37.2% (95% CI 31.6, 43.1) at baseline to 75.0% (95% CI 69.7, 79.6) in 2018 and 72.2% (95% CI 66.5, 77.2) in 2021. |
| Main results | 16 | (*a*) Give unadjusted estimates and, if applicable, confounder-adjusted estimates and their precision (eg, 95% confidence interval). Make clear which confounders were adjusted for and why they were included | 11-12 | Increases in completion rates were observed after implementation of the CHA program from baseline to 2018 (29.7 percentage points, 95% confidence interval (CI) [21.0, 38.4]), with maintenance but not further statistically significant increase from 2018 to 2021 (8.9pp [95% CI -0.1, 18.0]). Following CHA program implementation, the percentage of respondents who completed no steps of the continuum of care also decreased from 17.6% in 2015 to 4.0% (-12.4pp [95% CI -17.6, -7.2]) in 2018 and 3.7% (-12.6pp [95% CI -17.5, -7.7]) in 2021. |
|  |  | (*b*) Report category boundaries when continuous variables were categorized | N/A | N/A |
|  |  | (*c*) If relevant, consider translating estimates of relative risk into absolute risk for a meaningful time period | N/A | N/A |

Continued on next page

| Other analyses | 17 | Report other analyses done—eg analyses of subgroups and interactions, and sensitivity analyses | 13 | To account for the impact of the 2014 Liberian Ebola epidemic with much lower rates, sensitivity analyses were performed that removed births from 2014 while retaining 2015 births in the pre-implementation data set to not start with an abnormally low baseline. In order to maintain consistency, post-implementation responses in the immediate year preceding the other surveys were also removed (2017 births removed from 2018 survey responses, and 2020 births removed from 2021 survey responses), allowing for evaluation of results independent of the Ebola epidemic. Significant increases in full continuum of care completion and significant decreases in completion of no continuum of care steps after CHA implementation were still observed – see Table 3.  In multivariate regression models predicting completion of all steps in the maternal continuum of care (antenatal care, facility-based delivery, postnatal care), living between 10 and 20km from a health facility (odds ratio (OR) = 0.38, 95% confidence interval= [0.20, 0.71], p = < 0.01) and increased wealth (middle wealth quintile: OR = 2.32 [1.05, 5.15], p = 0.04; 4^th^ wealth quintile: OR = 2.72 [1.13, 6.58], p = 0.03; highest wealth quintile: OR = 2.29 [1.09, 4.81], p = 0.03) were associated with completing the full maternal continuum of care before implementation of the National Community Health Assistant Program. However, in follow up years, there was no longer a significant association between either distance or wealth with full continuum of care completion. Higher maternal education (OR = 0.57 [0.34, 0.93], p = 0.03) was the sole variable associated with decreased odds of completing the full maternal continuum of care at follow up (data not available at baseline). Prior to CHA program implementation, odds of completing no steps in the continuum of care was associated with increased distance from health care facilities (10-20 km away: OR = 1.94 [1.01, 3.71], p = 0.046; >20km away: OR = 3.09 [1.19, 8.02], p = 0.02) and increased maternal age (OR = 2.91 [1.10, 7.73], p = 0.03). Following implementation, only living at least 20 kilometers from a facility (OR = 7.64 [1.87, 31.26], p = < 0.01) was associated with absence of maternal care while no other variables were significant (Table 4). |
| --- | --- | --- | --- | --- |
| Discussion | | | | |
| Key results | 18 | Summarise key results with reference to study objectives | 15-16 | In this study, we found that implementation of the national CHA program in rural Liberia was associated with improvements in both completion of the full maternal continuum of care (reaching rates double that at baseline), as well as with reduction in women and their babies who had no formal care (reduced to less than 5% of births). … Our results contribute to existing knowledge of the value of community health worker programs but specifically address CHAs impact throughout the spectrum of maternal and early neonatal care utilization. |
| Limitations | 19 | Discuss limitations of the study, taking into account sources of potential bias or imprecision. Discuss both direction and magnitude of any potential bias | 16-17 | Our study has a number of limitations. We used retrospective self-reported uptake of the steps along the continuum of care, which could introduce opportunities for recall bias or overreporting of health care use due to social desirability. Importantly, we did not collect details on all components of the quality of care provided, nor on completion of the full scope of PNC. The sample sizes of each survey prevented our analyses from examining association of use of the continuum of care on infant and child mortality rates. Survey inconsistencies resulted in missing 2015 maternal education and birth characteristics. Because implementation covered the full county simultaneously, no comparison group was available—though the large effect size observed in this population is unlikely without other simultaneous large-scale health interventions. Finally, we did not include qualitative data collection to understand the barriers and facilitators to completion of steps and the full continuum of care. Additional studies with enough power to examine impacts on infant and child mortality rates as well as completion of essential newborn care are warranted for understanding how maternal counseling by CHAs can influence neonatal outcomes. |
| Interpretation | 20 | Give a cautious overall interpretation of results considering objectives, limitations, multiplicity of analyses, results from similar studies, and other relevant evidence | 17 | In conclusion, we found that implementation of the national CHA program was associated with a sustained increased in completion of maternal continuum of care and importantly, a reduction in mothers receiving none of the maternal continuum of care steps as well as reduction in distance and wealth as factors associated with gaps in care. This evidence points toward the capacity for community health assistant programs in rural areas to equitably drive participation in maternal and neonatal care that contribute to reducing maternal and neonatal mortality. Community-based interventions focused on delivering quality care to rural areas can succeed and can continue to be improved to fully reach all those in need. |
| Generalisability | 21 | Discuss the generalisability (external validity) of the study results | 16 | These results report specific outcomes related to one county in Liberia, but are similar to global review findings that CHA program implementations are successful at improving access to care at each individual stage in the continuum of care (19, 20). |
| Other information | |  | | |
| Funding | 22 | Give the source of funding and the role of the funders for the present study and, if applicable, for the original study on which the present article is based | 19 | Last Mile Health funded the data collection process. |
