# Supplementary material for: A repeated cross-sectional study of the association of community health worker intervention with the maternal continuum of care in rural Liberian communities
Source: BMC Pregnancy Childbirth. 2023 Dec 7;23:841. doi: 10.1186/s12884-023-06162-8 (PMC10701987; doi:10.1186/s12884-023-06162-8)
Supplement: Supplementary file 5 — Supplementary Material 5 [file 12884_2023_6162_MOESM5_ESM.docx]

Additional File 5: Appendix Table 1

**Appendix Table 1. Percentage of continuum of care completion for each possible combination of steps.**

| Group by combination | Continuum of care step(s) completed | | | Year | | |
| --- | --- | --- | --- | --- | --- | --- |
|  | ANC | FBD | PNC | 2015 | 2018 | 2021 |
| 1 |  |  |  | 17.6 (13.5, 22.8) | 4.0 (1.8, 8.3) | 3.7 (1.9, 6.8) |
| 2 | ✓ |  |  | 21.1 (16.2, 27.0) | 8.6 (5.5, 13.0) | 3.7 (2.1, 6.3) |
| 3 |  | ✓ |  | 5.8 (3.6, 9.0) | 3.0 (1.6, 5.3) | 4.3 (2.3, 8.1) |
| 4 |  |  | ✓ | 2.5 (1.4, 4.4) | 4.6 (2.6, 8.1) | 0 |
| 5 |  | ✓ | ✓ | 6.3 (4.0, 9.6) | 8.9 (6.4, 12.3) | 10.0 (7.2, 13.6) |
| 6 | ✓ |  | ✓ | 4.1 (2.5, 6.6) | 7.9 (5.3, 11.6) | 0 |
| 7 | ✓ | ✓ |  | 17.8 (13.6, 22.7) | 9.5 (6.6, 13.7) | 16.3 (12.3, 21.3) |
| 8 | ✓ | ✓ | ✓ | 25.0 (19.8, 31.2) | 53.6 (46.7, 60.4) | 62.1 (55.8, 68.1) |

All results are weighted, and percentages are reported with 95% confidence intervals.
