# Supplementary material for: A repeated cross-sectional study of the association of community health worker intervention with the maternal continuum of care in rural Liberian communities
Source: BMC Pregnancy Childbirth. 2023 Dec 7;23:841. doi: 10.1186/s12884-023-06162-8 (PMC10701987; doi:10.1186/s12884-023-06162-8)
Supplement: Supplementary file 6 — Supplementary Material 6 [file 12884_2023_6162_MOESM6_ESM.docx]

Additional File 6: Appendix Table 2

**Appendix Table 2. Change in PNC percentage rates over time, with most recent year of births removed.**

|  | 2015 | 2018 | 2021 | 2015-2018 change | P | 2015-2021 change | P | 2018-2021 change | P |
| --- | --- | --- | --- | --- | --- | --- | --- | --- | --- |
| Full maternal continuum of care | 27.0% | 54.4% | 62.0% | **27.3pp (15.3, 39.4)** | **<0.001** | **34.9pp (23.7, 46.2)** | **<0.001** | 7.6pp (-5.3, 20.5) | 0.25 |
| No steps in the maternal continuum of care | 15.1% | 2.9% | 2.8% | **-12.2pp (-19.2, -5.2)** | **<0.001** | **-12.3pp (-19.3, -5.3)** | **<0.001** | -0.1pp (-4.4, 4.2) | 0.95 |

Sensitivity analysis to evaluate outcomes with the time period of the 2014 Liberian Ebola epidemic removed. Results are reported for births that occurred within two years of surveys but not within one year (i.e., births from 2013 but not 2014 reported for 2015 survey, births from 2016 but not 2017 for 2018 survey, etc.).
